# Ginsenoside-Enriched Extract from Black Ginseng Anti-Fatigue Effects by Improving Antioxidant Capacity and Mitochondrial Function

**DOI:** 10.3390/life14111467

**Published:** 2024-11-12

**Authors:** Shunji Ge, Jiating Li, Xueyue Tai, Kuo Wang, Liyan Huang, Weixin Su, Guoqi Zhang, Bao Zhong, Fenglin Li

**Affiliations:** 1 College of Food Science and Nutritional Engineering, Jilin Agriculture Science and Technology University, Jilin 132101, China; shunjige2003@163.com (S.G.); 13351513723@163.com (X.T.); 17624189294@163.com (K.W.); 15144385407@163.com (L.H.); suweixinxx@163.com (W.S.); zhangguoqi@163.com (G.Z.); 2 School of Public Health, Jilin Medical University, Jilin 132013, China; m18291981180@163.com; 3 College of Food Science and Engineering, Changchun University, Changchun 130022, China; 4 School of Food Science and Engineering, Jilin Agricultural University, Changchun 130118, China; 5 College of Forestry, Beihua University, Jilin 132013, China

**Keywords:** black ginseng, ginsenosides, anti-fatigue, antioxidant, mitochondrial

## Abstract

In this study, we investigated the anti-fatigue effects of black ginseng ginsenosides using exercise performance tests, serum analyses, and gene expression profiling. No significant differences in dietary intake or body weight were observed between groups. The low-dose black ginseng (LBG) group showed no significant improvements in swimming and rotating rod tests. In contrast, the medium (MBG)- and high-dose (HBG) groups showed notable increases in swimming time and significant improvements in the rotating rod test. All treatment groups exhibited longer running times, particularly the HBG group. Serum analysis revealed increased muscle and hepatic glycogen, catalase, and lactate dehydrogenase levels in the MBG and HBG groups, whereas lactate, lipid peroxide, and superoxide dismutase levels were decreased. Additionally, gene expression analysis showed significant upregulation of key antioxidant and mitochondrial function genes, including those encoding cationic amino acid transporter 2, stearoyl-CoA desaturase-2, nuclear respiratory factor 1, nuclear factor erythroid 2-related factor 2, mitochondrial transcription factor A, cytochrome c oxidase II, and NADH–ubiquinone oxidoreductase core subunit 1, particularly in the HBG group, indicating enhanced antioxidant capacity and improved mitochondrial function. These findings suggested that black ginseng ginsenosides effectively mitigated fatigue.

## 1. Introduction

Ginseng (*Panax ginseng* C. A. Meyer) is a slow-growing herbaceous plant belonging to the *Araliaceae* family and the Panax genus. The genus name “Panax” is derived from the Greek word “panacea”, meaning “a remedy for all diseases”. There are 200 types of saponins in ginseng, which can be divided into three categories: protopanaxadiol, protopanaxatriol, and oleanane [1]. Ginsenosides exert pharmacological effects in anti-inflammation, anti-cancer, cardiovascular protection, immune regulation, neuroprotection, anti-diabetes, anti-fatigue, anti-allergy, anti-aging, and antioxidant aspects [2]. The potential reason why ginsenoside solution can counteract fatigue is because it regulates the expression of liver glycogen, muscle glycogen, hepatocyte malondialdehyde (MDA), and hepatocyte superoxide dismutase (SOD) in the blood [3]. 20 (R)-ginsenoside Rg3 exhibits a regulatory effect on fatigue by regulating the expression of serum urea nitrogen (SUN), lactate dehydrogenase (LDH), SOD, MDA, blood lactic acid (LA), and liver glycogen [4].

Unlike its conventional counterpart, black ginseng is a new product formed by ginseng undergoing a unique processing method, involving steaming and drying that causes extensive changes in the type and amount of secondary metabolites [5]. Black ginseng has garnered significant attention in herbal medicine because of its diverse pharmacological properties. There are abundant chemical components in black ginseng, including ginsenosides, polysaccharides, amino acids, polyphenols, and flavonoids [6]. Among these, ginsenosides, polysaccharides, and phenolic compounds are the main ingredients making the health benefits of black ginseng stronger than those of other ginseng products. Research has shown that black ginseng contains rarer ginsenosides, such as Rg3, Rk1, and Rg5, than white ginseng [7]. These unique compounds are believed to contribute to the health benefits of black ginseng. Furthermore, some Maillard reaction products, such as maltol, have also been reported [8]. Black ginseng is associated with a noticeable superiority over white and red ginseng in most comparative biological studies due to its enhanced bioactive components, particularly ginsenosides [9]. Thus, the distinct preparation method of black ginseng is believed to improve its efficacy and alter the pharmacokinetics of its bioactive constituents, resulting in enhanced therapeutic potential [10]. The efficacy of black ginseng has been the subject of numerous studies, which have shown that black ginseng enhances immune function by promoting the proliferation of immune cells and increasing the production of cytokines, thereby bolstering defense mechanisms [11]. Moreover, it improves cognitive performance and exerts anti-fatigue effects [12]. Additionally, its antioxidant properties play a crucial role in reducing the oxidative stress associated with various chronic diseases [13]. Fatigue is a complex syndrome affecting both physical and cognitive function [14]. Fatigue is characterized by decreased physical and mental performance, often resulting from prolonged exertion or stress [15]. A significant body of research has specifically addressed the anti-fatigue properties of black ginseng. The mechanisms underlying these benefits are attributed to the modulation of various biological pathways, including the regulation of oxidative stress and inflammation, which are critical factors in fatigue development [16]. Studies have demonstrated that black ginseng can alleviate fatigue through multiple mechanisms. It enhances energy metabolism by increasing ATP production in muscle tissues [17]. Additionally, ginseng exhibits potent antioxidant activity, which helps mitigate oxidative damage induced by physical exertion, thereby improving endurance and recovery [18]. In animal models, supplementation with black ginseng results in improved exercise performance and reduced lactate levels and muscle damage and other biomarkers of fatigue [19,20].

The *CAT-2* (cationic amino acid transporter 2) and *Scd-2* (stearoyl-CoA desaturase-2) signaling pathways have emerged as crucial mediators in various physiological processes, including the regulation of oxidative stress and energy metabolism, both of which are critical in the context of fatigue [21]. *CAT-2* plays a significant role in the transport of amino acids, particularly arginine, which is vital for the synthesis of nitric oxide (NO) [22]. NO is a key signaling molecule involved in various physiological processes, including vasodilation and the modulation of mitochondrial function. Enhanced NO production can improve blood flow and oxygen delivery to muscles during physical activity, thereby reducing fatigue [23,24]. *Scd-2*, on the other hand, is involved in lipid metabolism, particularly in the synthesis of monounsaturated fatty acids from saturated fatty acids [25]. This process is crucial for maintaining cellular membrane integrity and function and can be compromised by prolonged exercise or stress. By promoting the synthesis of monounsaturated fatty acids, Scd-2 can improve mitochondrial function and energy production, contributing to increased stamina and reduced fatigue [26]. *NRF1* (nuclear respiratory factor 1) and *NRE2L2* (nuclear factor erythroid 2–related factor 2) are key regulators of mitochondrial biogenesis and oxidative stress responses [27,28]. *NRF1* primarily facilitates the transcription of genes involved in mitochondrial respiration and biogenesis, including those that encode components of the electron transport chain. Enhanced NO production from the *CAT-2* pathway can stimulate mitochondrial biogenesis via *NRF1*, supporting energy production and reducing fatigue [29,30]. In contrast, *NRE2L2* is crucial for the cellular antioxidant response by promoting the expression of antioxidant enzymes that protect against oxidative damage [31]. Together, these factors help maintain mitochondrial health and function, which are vital for sustaining energy levels during physical exertion. *TFAM* (mitochondrial transcription factor A) is a critical transcription factor that directly regulates the expression of mitochondrial DNA, including genes essential for oxidative phosphorylation [32,33]. By promoting mitochondrial DNA replication and transcription, *TFAM* ensures the production of key mitochondrial proteins, including mitochondrial cytochrome c oxidase II (*MtCOX2*) and mitochondrial NADH–ubiquinone oxidoreductase core subunit 1 (*MtND1*), which are involved in the electron transport chain [34]. Additionally, lipid profiles regulated by Scd-2 may influence mitochondrial function and are essential for the optimal expression of *NRF1*, *NRE2L2*, and *TFAM*.

Given the increasing prevalence of fatigue-related diseases in modern society, it is crucial to understand the mechanisms by which black ginseng exerts its anti-fatigue effects. This study aimed to elucidate the specific pathways involved in the anti-fatigue effects of black ginseng ginsenosides and their mechanism(s) of action.

## 2. Materials and Methods

### 2.1. Preparation of Freeze-Dried Black Ginseng Ginsenoside Powder

Fresh ginseng (Fusong County, Jilin Province, China), approximately 1.5 cm in diameter, was thoroughly washed and drained. The roots were dried at 60 °C for 8 h, followed by steaming at 121 °C for 2 h and simmering for 1 h. The ginseng was dried again at 60 °C to produce black ginseng. To prepare the extract, black ginseng powder was mixed with a 70% ethanol solution at a solid-to-liquid ratio of 1:40 (g/mL). The mixture was subjected to ultrasonic treatment for 30 min with ultrasonic power exceeding 500 W (XH-300A Ultrasonic Extractor, Beijing Xianghu Technology Development Co., Ltd., Beijing, China). Following sonication, the mixture was centrifuged, and the supernatant was collected. Finally, the supernatant was freeze-dried to obtain black ginseng ginsenoside powder.

### 2.2. Determination of Black Ginseng Ginsenoside Components by High-Performance Liquid Chromatography

Stock solutions of ginsenoside standards, mixtures including Rg1, Re, Rb1, RC, Rb2, S-Rg2, R-Rg2, Rh1, Rd, S-Rg3, R-Rg3, RK1, and Rg5 were prepared to assess the retention time and content of saponins. High-performance liquid chromatography (HPLC) analysis was conducted at a flow rate of 0.8 mL/min, a column temperature of 35 °C, and a detection wavelength of 203 nm, with acetonitrile and water serving as the mobile phases. The saponin components of black ginseng were analyzed via HPLC. The chromatographic conditions included a C18 column (4.6 mm × 150 mm), a UV detector set to a detection wavelength of 203 nm, an acetonitrile mobile phase, a flow rate of 0.3 mL/min, and an injection volume of 10 µL. A gradient elution program was established, starting with 90% A (water) and 10% B (acetonitrile), which was gradually transitioned to 50% A and 50% B over 30 min, followed by a return to the initial conditions for re-equilibration. The flow rate during the analysis was set to 1.0 mL/min, allowing the determination of saponin content.

### 2.3. Animal Experiment

A total of 60 male ICR mice (weighing approximately 20 ± 2 g, sourced from Changchun Yisi Experimental Animal Technology Co., Ltd., Changchun, China) were used in this study. The mice were housed in an environment maintained at a temperature of 23 ± 2 °C and a humidity of 55 ± 5%, with a 12 h light/dark cycle. They were provided with a standard diet and had free access to water. After a 7-day acclimatization period, the mice were randomly assigned to one of four groups based on their body weight: a control group, low-dose black ginseng ginsenosides (LBG, 300 mg/kg), medium-dose black ginseng ginsenosides (MBG, 400 mg/kg), and high-dose black ginseng ginsenosides (HBG, 500 mg/kg) groups. The mice received daily gavage administration for 21 d, with body weights and other data recorded every 2 d. The daily food intake was also monitored. Starting on day 15, the mice underwent adaptation training, which included treadmill running, swimming, and rotating rod exercises (Jiangsu Saiangsi Biotechnology Co., Ltd., Taizhou, China). On day 21, the mice were fasted before being subjected to the treadmill, swimming, and rotating rod tests. The running time, swimming endurance time, and rotating rod duration were recorded. Following these experiments, the mice were euthanized, and blood samples (from which serum was obtained following blood clotting via centrifugation), muscle tissue, and liver were collected and stored at −80 °C for further analysis.

### 2.4. Determination of Blood Biochemical Parameters

Serum biochemical parameters were determined following the instructions provided with the respective assay kits. The analysis included the measurement of muscle glycogen (MG), hepatic glycogen (HG), lactate, lactate dehydrogenase (LDH), catalase, lipid peroxides, and total superoxide dismutase (T-SOD) levels. All test kits were obtained from the Nanjing Jiancheng Bioengineering Institute.

### 2.5. Anti-Fatigue Gene PCR Expression

Total RNA was extracted from the liver using a TRIzol reagent, and the extracted RNA was quantified using a NanoDrop (Thermo Fisher, Waltham, MA, USA). The cDNA was synthesized using the FastKing cDNA First Strand Synthesis Kit, and quantitative PCR (qPCR) was performed using SYBR Green qPCR mixture (Tiangen Biochemical Technology Co. Beijing, China). PCR thermocycling conditions were denaturation at 95 °C for 15 s, annealing at 60 °C for 20 s, and extension at 72 °C for 35 s, with a final extension at 72 °C for 35 s for a total of 45 cycles. The Primer sequences for the genes are listed in Table 1.

### 2.6. Statistical Analysis

All values are presented as mean ± standard deviation. The differences between the groups were assessed using Duncan’s multiple comparison test with SPSS ANOVA software (SPSS Statistics 23; IBM Corp., Armonk, NY, USA; *p* < 0.05). Significant results among the groups are indicated by the letters a, b, c, and d, with the relationship: a > b > c > d.

## 3. Results

### 3.1. HPLC Analysis of Black Ginseng Ginsenoside Content

**Figure 1:**
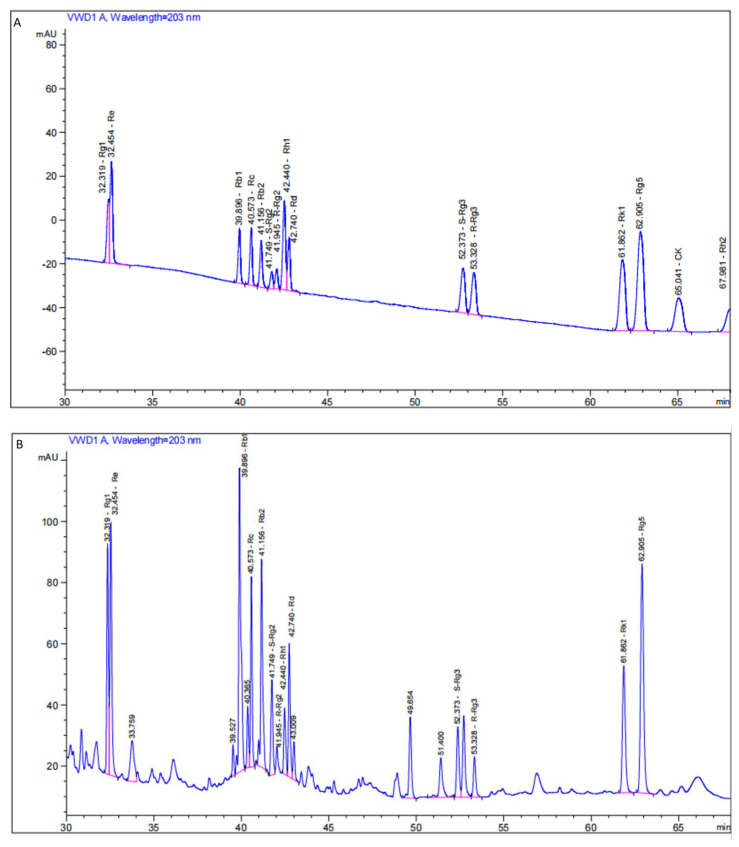
High-performance liquid chromatography profiles. (**A**) High-performance liquid chromatography profiles of ginsenoside standards; (**B**) high-performance liquid chromatography profiles of ginsenosides in black ginseng.

The determination of black ginseng ginsenoside content is presented in Figure 1 and Table 2. Thirteen ginsenosides were identified based on their retention times compared to standards. The concentrations of these ginsenosides in black ginseng (values in brackets in μg/mL) were as follows: Rg1 (41.22), Re (61.80), Rb1 (122.03), Rc (50.14), Rb2 (55.49), S-Rg2 (45.90), R-Rg2 (14.44), Rh1 (9.59), Rd (24.25), S-Rg3 (158.84), R-Rg3 (63.97), RK1 (98.89), and Rg5 (92.69).

### 3.2. Impact on Exercise Training in Mice

The weight, food intake, and exercise test results of the mice during the experiment are summarized in Table 3. No significant differences were observed in the dietary intake or weight between the groups throughout this study. However, variations in exercise performance were observed with different doses of black ginseng ginsenoside supplementation. In the swimming test, the LBG did not show a significant effect compared to the control group, whereas both the MBG and HBG groups exhibited a notable increase in swimming time. In the rotating rod test, no significant differences were found between the LBG and control groups. In contrast, the MBG and HBG groups demonstrated a significant improvement in rod performance compared with the control group. The results of the running experiment indicated that the LBG, MBG, and HBG groups had significantly increased running times compared with the control group, with high-dose black ginseng ginsenoside supplementation producing the most pronounced effect on enhancing running endurance.

### 3.3. Serum Index Biological Parameters

**Figure 2:**
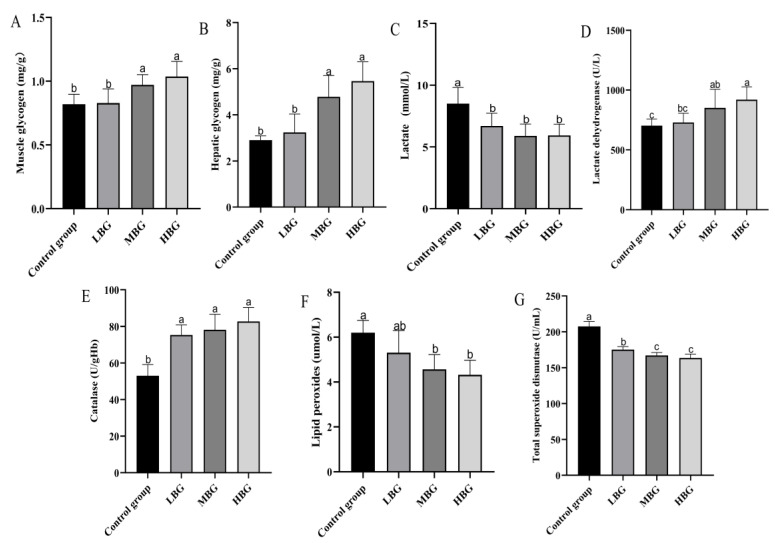
The effect of black ginseng ginsenoside treatment on the tissue and serum markers of fatigue in mice. (**A**) Muscle glycogen, (**B**) hepatic glycogen, (**C**) lactate, (**D**) lactate dehydrogenase, (**E**) catalase, (**F**) lipid peroxides, and (**G**) total superoxide dismutase. The animals were gavaged daily for 21 d with either a low—(LBG group, 300 mg/kg), medium—(MBG group, 400 mg/kg), or high—(HBG group, 500 mg/kg) dose of black ginseng ginsenoside extract. Following an exercise challenge involving treadmill running, swimming, and rotating rod exercises, the mice were killed, and serum samples were obtained. The results are the mean ± standard deviation. The values bearing different superscripted letters are significantly different.

The effects of black ginseng ginsenosides on biomarkers of fatigue in mice are shown in Figure 2. Compared with the control group, muscle and hepatic glycogen levels increased significantly in the MBG and HBG groups but not in the LBG group. Compared with the control group, serum lactate levels decreased significantly in each of the black ginseng ginsenoside doses tested. Conversely, serum LDH activity significantly increased in the MBG and HBG groups compared to that in the control group. Serum catalase activity was significantly increased at all three black ginseng saponin doses tested. Compared to the control group, the MBG and HBG groups showed significant regulatory effects on lipid peroxide levels in serum. Compared with the control group, the LBG, MBG, and HBG groups showed significantly decreased serum T-SOD levels, with the effect being larger in the MBG and HBG groups.

### 3.4. Real-Time PCR Analysis

**Figure 3:**
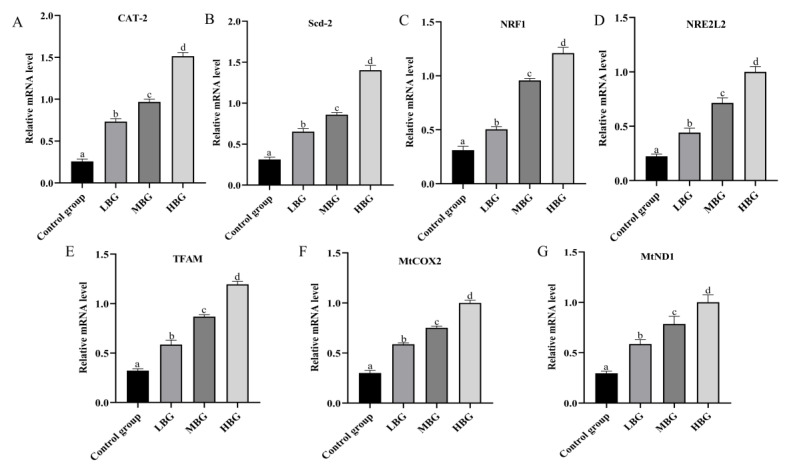
The expression of hepatic genes involved in fatigue determined by real-time PCR. The relative mRNA levels are presented as the mean ± standard deviation. The values bearing different superscripted letters are significantly different. (**A**) *CAT-2*, cationic amino acid transporter 2; (**B**) Scd-2, stearoyl-CoA desaturase-2; (**C**) *NRF1*, nuclear respiratory factor 1; (**D**) *NRE2L2*, nuclear factor erythroid 2–related factor 2; (**E**) *TFAM*, mitochondrial transcription factor A; (**F**) *MtCOX2*, cytochrome c oxidase II; and (**G**) *MtND1*, mitochondrial NADH–ubiquinone oxidoreductase core subunit 1.

The results of the gene analysis are shown in Figure 3. Compared to the control group, the expression levels of *CAT-2* and *Scd-2* in the LBG, MBG, and HBG groups were significantly increased, with the greatest effects occurring in the HBG. Compared to the control group, the expression levels of *NRF1*, *NRE2L2*, *TFAM*, *MtCOX2*, and *MtND1* in the LBG, MBG, and HBG groups were significantly increased. The HBG group exhibited a more significant effect on the regulation of *NRF1*, *NRE2L2*, *TFAM*, *MtCOX2*, and *MtND1* than the LBG and MBG groups.

## 4. Discussion

This study evaluated the effects of black ginseng ginsenosides on exercise performance through swimming, running, and rotating rod experiments. Notably, while the group did not demonstrate significant differences in swimming performance compared to the control group, both the MBG and HBG groups showed marked improvements in swimming time. This aligns with previous research suggesting that saponins may enhance endurance by increasing energy metabolism and reducing fatigue [35,36]. In the rotating rod test, the LBG group did not differ significantly from the control group, whereas the MBG and HBG groups performed significantly better. This finding indicates that higher doses of black ginseng ginsenosides may be necessary to effectively enhance motor coordination and resistance to fatigue [37]. Similarly, the running experiment revealed that all three doses of black ginseng ginsenosides significantly increased running times relative to the control group, with the HBG group demonstrating the most pronounced effect. This suggests a dose-dependent relationship between the fatigue-reducing properties of black ginseng ginsenosides, supporting the notion that higher concentrations may optimize athletic performance. Overall, these results indicate that black ginseng ginsenosides, particularly at medium-to-high doses, can effectively enhance exercise performance across different modalities.

Serum biochemical analysis highlighted the regulatory effects of black ginseng ginsenosides on various indicators of fatigue in mice. Notably, while the LBG group did not show significant changes in muscle and hepatic glycogen levels compared with the control group, both the MBG and HBG groups demonstrated substantial increases in these parameters. Elevated glycogen levels suggest enhanced energy availability during exercise, which potentially improves endurance [38].

Furthermore, serum lactate levels significantly decreased in the LBG, MBG, and HBG groups, indicating a reduction in anaerobic metabolism and muscle fatigue [39,40]. LDH is a marker of metabolic stress and was notably increased in the MBG and HBG groups, reinforcing the protective effects of black ginseng ginsenosides against exercise-induced fatigue [32,41,42]. Additionally, the significant increase in catalase levels across all black ginseng ginsenoside-treated groups suggests an enhanced antioxidant defense mechanism, which is crucial for mitigating the oxidative stress associated with prolonged physical activity [43]. The regulation of lipid peroxide levels by MBG and HBG indicates a reduction in oxidative damage, further supporting the protective role of black ginseng ginsenosides against fatigue. Finally, T-SOD levels were significantly improved in all treatment groups, with MBG and HBG exhibiting the most pronounced effects. This indicates that the upregulation of antioxidant enzymes contributes to the overall reduction in oxidative stress and fatigue during exercise [44]. Collectively, these findings suggest that black ginseng ginsenosides effectively enhance exercise performance and reduce fatigue through multiple biochemical pathways.

NO levels are critical during skeletal muscle contraction. Changes in NO levels play a role in muscle fatigue [45]. Studies have shown that *CAT-2* plays an important role in inhibiting NO production [46]. *Scd-2* plays essential roles in regulating lipid metabolism and maintaining mitochondrial function, both of which are vital for energy production. The observed increase in *CAT-2* and *Scd-2* expression in the LBG, MBG, and HBG groups suggests that black ginseng ginsenosides enhance cellular antioxidant capacity and improve mitochondrial function, which may reduce oxidative damage during strenuous exercise [47,48]. Notably, the HBG group exhibited the most pronounced effect, indicating a dose-dependent relationship that aligns with enhanced anti-fatigue properties. *NRF1* and *NRE2L2* are transcription factors that regulate the expression of genes involved in antioxidant defense and mitochondrial biogenesis. The activation of *NRE2L2* leads to the upregulation of various antioxidant enzymes, thereby enhancing the cellular response to oxidative stress [49,50]. *TFAM* is essential for mitochondrial DNA replication and transcription, whereas *MtCOX2* and *MtND1* are critical components of the mitochondrial respiratory chain that play key roles in ATP synthesis. Our results showed that black ginseng ginsenosides significantly increased the expression of *NRF1*, *NRE2L2*, *TFAM*, *MtCOX2*, and *MtND1* in all treatment groups, with the HBG group showing the most substantial regulatory effects. This suggests that ginseng saponins promote mitochondrial biogenesis and enhance oxidative phosphorylation, leading to improved energy production and reduced fatigue [51,52]. The enhanced antioxidant capacity of *CAT-2* and *Scd-2* contributes to a favorable environment for mitochondrial function, which is further supported by the activation of *NRF1* and *NRE2L2*. This synergy ensures that cellular energy production is optimized, oxidative stress is minimized, and the overall muscle performance is improved. In conclusion, the anti-fatigue effects of black ginseng ginsenosides are mediated by the activation of both the *CAT-2/Scd-2* and *NRF1/NRE2L2/TFAM/MtCOX2/MtND1* signaling pathways. These findings provide valuable insights into the biochemical mechanisms underlying the health benefits of black ginseng ginsenosides and highlight their potential as therapeutic agents to enhance physical performance and combat fatigue.

The results from animal model studies provide valuable insights into potential implications for human metabolism. While there are inherent differences between species, animal studies often serve as a preliminary platform for understanding complex biological mechanisms. In the context of our research on black ginseng ginsenosides, the demonstrated anti-fatigue effects, coupled with improvements in antioxidant capacity and mitochondrial function, suggest several relevant implications for human health. Animal models have shown that oxidative stress plays a significant role in fatigue and metabolic disorders [53]. By improving antioxidant capacity, black ginseng ginsenosides may mitigate oxidative damage, reducing fatigue in humans, especially in conditions of physical stress or chronic fatigue syndrome. Mitochondria are crucial for energy production and metabolic health. Enhancements in mitochondrial function observed in our animal studies may indicate similar benefits for human subjects. For instance, improved mitochondrial biogenesis and function can lead to better energy metabolism, potentially aiding in exercise performance and recovery [54].

## 5. Conclusions

This study examined the anti-fatigue effects of black ginseng ginsenosides using exercise performance tests, serum analyses, and gene expression profiling. In the exercise tests, no significant differences in dietary intake or body weight were observed between the groups. The LBG group did not show significant improvements in the swimming test compared to the control group, whereas both the MBG and HBG groups demonstrated notable increases in swimming time. There were significant improvements in the MBG and HBG groups compared to the control group in the rotating rod test. The LBG, MBG, and HBG groups showed significantly longer running times, with the HBG group showing the most pronounced effect on endurance. Serum analysis indicated significant increases in muscle and hepatic glycogen levels in the MBG and HBG groups compared to those in the control group, along with decreased lactate, lipid peroxides, and T-SOD levels. Additionally, the MBG and HBG groups showed significantly increased LDH and catalase levels, highlighting the enhanced antioxidant activity. Gene expression analysis revealed a significant upregulation of *CAT-2*, *Scd-2*, *NRF1*, *NRE2L2*, *TFAM*, *MtCOX2*, and *MtND1* in all treatment groups, with the HBG group exhibiting the strongest regulatory effects, indicating improved mitochondrial function and antioxidant capacity. While this study presents compelling evidence for the anti-fatigue effects of black ginseng ginsenosides through enhanced antioxidant capacity and improved mitochondrial function, several limitations must be acknowledged. The reliance on animal models may restrict the generalizability of our findings, underscoring the need for future clinical trials. Additionally, although this research concentrated on biochemical and physiological parameters, the potential impact of black ginseng ginsenosides on cognitive fatigue and psychological well-being warrants further exploration. Incorporating subjective assessments of fatigue and quality of life in future studies could yield a more comprehensive understanding of the effects of ginsenosides.

## Figures and Tables

**Table 1:** The primer sequence of the genes.

Gene	Forward Primer	Reverse Primer
*CAT-2*	ATCTTCTCCATCGTGGGCTTCAT	CCATGGATATGTGTACTTC
*Scd-2*	TGGTGCCCTGGTACTGCT	CGATGAAGAACGTGGTGAAG
*NRF1*	GGCGGGAGGACCTTCTGTATGC	GGCCCAATTTTGTTCCACCTCTCC
*NRE2L2*	CAGCCCAGCACATCCAGACAGA	GAATATCCAGGGCAAGCGACTCAT
*TFAM*	CGGGGAATGTGGGGCGTGCTAA	GCGTTTCTGCCGGGCTTCCTTCTC
*MtCOX2*	ACAAGACGCCACATCACCTATCAT	TACTTCTTGGGCGTCTATTGTGCT
*MtND1*	GCAAAGGCCCCAACATCGTAG	TAAGGGGGTGAGGTATTGGTAAGG

**Table 2:** Retention time and content of black ginseng ginsenosides.

No.	Name	Retention Time (min)	Content (μg/mL)
1	Rg1	32.319	41.21893 ± 0.068
2	Re	32.454	61.79905 ± 0.049
3	Rb1	39.896	122.02576 ± 0.042
4	RC	40.573	50.13992 ± 0.015
5	Rb2	41.156	55.49115 ± 0.022
6	S-Rg2	41.749	45.89925 ± 0.029
7	R-Rg2	41.945	14.43858 ± 0.003
8	Rh1	42.440	9.59264 ± 0.003
9	Rd	42.740	24.24622 ± 0.002
10	S-Rg3	52.392	158.83643 ± 0.062
11	R-Rg3	53.009	63.97367 ± 0.041
12	RK1	61.862	98.88668 ± 0.046
13	Rg5	62.905	92.69036 ± 0.013
Total			839.23864 ± 0.212

**Table 3:** The results of the feeding, body weight, and exercise experiment on the mice supplemented with black ginseng ginsenosides.

Groups	Food Intake (g/d)	Final Weight (g)	Swimming Time (min)	Rotating Rod Time (s)	Running Time (s)
Control group	5.33 ± 0.26 ^a^	33.32 ± 1.92 ^a^	17.17 ± 1.28 ^b^	1371.04 ± 41.25 ^c^	809.00 ± 33.80 ^d^
LBG	5.31 ± 0.18 ^a^	32.56 ± 2.11 ^a^	17.49 ± 0.43 ^b^	1432.30 ± 30.67 ^c^	1344.00 ± 51.53 ^c^
MBG	5.29 ± 0.18 ^a^	32.54 ± 2.61 ^a^	21.06 ± 0.69 ^a^	1723.84 ± 84.77 ^b^	1777.00 ± 49.09 ^b^
HBG	5.36 ± 0.28 ^a^	32.68 ± 1.89 ^a^	21.97 ± 0.91 ^a^	1812.70 ± 67.16 ^a^	2095.00 ± 67.16 ^a^

The results are the mean ± standard deviation (15 mice per group). Control group; LBG, low-dose black ginseng ginsenoside group (300 mg/kg); MBG, medium-dose black ginseng ginsenoside group (400 mg/kg); HBG, high-dose black ginseng ginsenoside group (500 mg/kg). The values within a column bearing different superscripted letters are significantly different.

## Data Availability

The data presented in this study are available on request from the corresponding author.
